# Parasites Found in the Vermiform Appendix, with Report of a Case

**Published:** 1896-10

**Authors:** H. T. Williams

**Affiliations:** Rochester, N. Y.; Surgeon to Rochester City Hospital and St. Mary’s Hospital, Rochester, N. Y.


### PARASITES FOUND IN THE VERMIFORM APPENDIX, WITH REPORT OF A CASE.

By H. T. WILLIAMS, M. D., Rochester, N. Y.,
Surgeon to Rochester City Hospital and St. Mary's Hospital, Rochester, N. Y.

LAST October I operated upon a case of appendicitis, of which the exciting cause, at least, proved to be the presence of a living worm in the vermiform appendix. The history of the case is as follows :

The patient, a young married woman, who had always enjoyed excellent health, was attacked in the night with sudden severe pain in her abdomen, more severe on the right side, and extending to the back. Temperature normal, but pulse rapid ; no special tenderness on pressure over McBurney's area. There was, however, considerable vesicle tenesmus. The attack was ascribed to a cold. The next morning she was better and went out of doors, but in the evening was taken with sudden severe pain in the right iliac region. Temperature, 99 ; pulse, 130 to 140. Large doses of morphia were required to subdue the pain. Persistent pain and vomiting all the next day, and great tenderness over McBurney's point. Bowels moved freely several times. Temperature, 101 2-5 in the morning, rose to 103 2-5 in the evening. The patient was removed to the City hospital and operation performed late that night. After making the usual incision and introducing a finger into the abdominal cavity, some slight adhesions were found around the appendix, which were easily broken up, and the appendix, which was highly congested, swollen and filled with a dark fluid and some pus, was removed, the stump being encircled with a silk ligature and the opening touched with a cautery point. The edges were then turned in and the peritoneal covering stitched with fine cat-gut and the abdominal opening closed in the usual way.

The patient made an uneventful recovery, was out of bed in ten days, and left the hospital in two and one-half weeks. In the extreme end of the appendix, surrounded with pus and liquid feces, was found a living worm, about three-eighths of an inch in length. It proved to be a female oxyuris vermicularis, commonly called thread-worm. The appendix was very much thinned at this point, where the worm was found, and it was very evident that a perforation would soon have occurred. As I had never heard of a similar case before, I was interested to learn if these or other parasites were frequently found alive in the vermiform appendix; but, after much investigation, I have not been able as yet to find a similar case. It is said the ascaris lumbricoides have been found dead in the appendix, but no living worm of any kind.

Keen and DaCosta make the assertion that living worms are never found in the vermiform appendix. When we consider that the true habitat of the oxyuris vermicularis is in the cecum, (the general belief that they reside in the rectum and sigmoid flexure, because they migrate there in large numbers, is erroneous,) and that they have a tendency to migrate, it is to be wondered at that they do not frequently wander into the appendix and cause trouble.

It may not be uninteresting, although not strictly in line of my subject, to state that in my investigations into the literature of appendicitis I have been surprised to find the variety of foreign bodies reported as having been found in the appendix—pins, fish-bones, glass beads, buttons, fragments of straw, nails, screws, cherry stones, pear, grape, melon, apple and orange seeds, beans, date, prune and peach stones, gall-stones, toilet pins and other foreign bodies too numerous to mention.

Dr. William T. Bull reports thirteen cases in which nothing but pus and mucus was found.

Fecal concretions, single and multiple, and of various shapes, sizes and consistence, are most commonly found, and are believed by most writers to be the exciting cause of appendicitis in most cases.

The bacteriological studies of the contents of the vermiform appendix seem to prove the presence there of the bacillus coli communis, the bacillus pyogenes fetidus and occasionally a streptococcus. What part the bacillus coli communis plays in the etiology of appendicitis I am not prepared to say, since Hodenpyle found the microbe in pure cultures in five normal appendices. Dr. Maurice H. Richardson, in his report of a personal experience of 181 cases of appendicitis, states that the bacillus was found in all cultures made from the fluids found in the appendix in severe cases, and especially the so-called fulminating cases, but did not find it in many mild cases. Azoulay considers "appendicitis to be a form of septic inflammation, clinically and pathologically, the agent being probably the bacillus coli communis, sometimes aided by other bacteria, making the infection complex." He suggests as an explanation of the virulence of a microbe normally found in the intestines, that the epithelium is injured by fecal masses or other foreign bodies, thus removing the barrier to the entrance of the microbe into the adenoid tissue, in which situation they rapidly originate an infective inflammation.

